# Age at diagnosis of diabetes, obesity, and the risk of dementia among adult patients with type 2 diabetes

**DOI:** 10.1371/journal.pone.0310964

**Published:** 2024-11-13

**Authors:** Xiang Qi, Zheng Zhu, Huabin Luo, Mark D. Schwartz, Bei Wu

**Affiliations:** 1 Rory Meyers College of Nursing, New York University, New York, New York, United States of America; 2 School of Nursing, Fudan University, Shanghai, China; 3 Brody School of Medicine, East Carolina University, Greenville, North Carolina, United States of America; 4 Grossman School of Medicine, New York University, New York, New York, United States of America; National Research Centre, EGYPT

## Abstract

**Background:**

While Type 2 Diabetes Mellitus (T2DM) prevalence is increasing among younger individuals, few studies have examined how age at T2DM diagnosis relates to dementia risk in diabetic populations. We aimed to investigate the association between age at T2DM diagnosis and subsequent dementia risk, and to determine whether obesity moderates this relationship.

**Methods:**

We conducted a prospective cohort study using data from the Health and Retirement Study (2002–2016) matched with its 2003 Diabetes Mail-Out Survey. The study included 1,213 dementia-free adults aged ≥50 with diagnosed T2DM. Primary exposures were age at T2DM diagnosis (categorized as <50, 50–59, 60–69, and ≥70 years) and obesity status (BMI ≥30 kg/m^2^). The outcome was incident dementia, assessed using the Telephone Interview for Cognitive Status. Cox proportional hazards models were used to estimate hazard ratios (HRs) and 95% confidence intervals (CIs), adjusting for sociodemographic factors, health behaviors, health status, and diabetes medication use.

**Results:**

Over a median follow-up of 10 (interquartile range, 6–14) years, 216 (17.8%) participants developed dementia. Compared to participants diagnosed with T2DM at age ≥70 years, those diagnosed at younger ages had increased dementia risk: HR 1.70 (95% CI, 1.03–2.80) for 60–69 years, 1.72 (95% CI, 1.06–2.79) for 50–59 years, and 1.90 (95% CI, 1.14–3.18) for <50 years. Obesity significantly moderated this relationship, with obese individuals diagnosed with T2DM before age 50 showing the highest dementia risk (HR 3.05; 95% CI 1.23–7.56) compared to non-obese individuals diagnosed at ≥50 years.

**Conclusions:**

Younger age at diagnosis of T2DM was significantly associated with a higher risk of dementia, particularly among individuals with obesity. Interventions specifically targeting obesity may be more effective in preventing dementia for adults with a younger onset of T2DM.

## Introduction

Dementia has become a major threat to global public health with population aging. In 2021, over 6.5 million older Americans were living with dementia, and the total cost for individuals with dementia is $355 billion in the US [1]. Diabetes mellitus, affecting 29.2% of older adults in the US [2, 3], has been linked to a higher risk of dementia (1.43-fold excess risk) [4, 5]. According to the International Diabetes Federation (IDF) Diabetes Atlas (10th edition, 2021), approximately 6.7 million people aged 65 years or older with diabetes have dementia globally [6]. Based on the results from a meta-analysis, the age-standardized prevalence of dementia in people with diabetes aged 60 years or older is 13.5% in North America and Europe, compared to 7.9% in South Asia and 6.7% in Sub-Saharan Africa [7]. As of 2022, approximately 16.0% of Americans aged 65 and older with diabetes also have a diagnosis of Alzheimer’s disease or other dementias [8]. While high-income countries currently have higher prevalence rates, the rate of increase is expected to be more rapid in low- and middle-income countries due to demographic transitions and increasing diabetes prevalence [9]. Although the underlying mechanisms remain unclear, it has been hypothesized that cerebrovascular pathology is affected by hyperglycemia and insulin resistance. Moreover, specific treatment for dementia in individuals with type 2 diabetes mellitus (T2DM) has been unknown. Gaining a better understanding of risk factors for dementia is critical for determining how interventions may be developed to prevent dementia in individuals with T2DM [4].

T2DM accounts for more than 90% of all diabetes cases. Although T2DM was conventionally recognized as a disease of older adults, a rapid prevalence increase of T2DM has been observed among individuals at younger ages [10]. Globally, 20% of individuals with diabetes were diagnosed before 40 years (i.e., early-onset of T2DM) [10]. Recent studies have shown that individuals with early-onset T2DM have worse dementia risk factor profiles (body mass index [BMI], glycemia levels, and lipids) relative to those with late-onset [11], and increased cardiovascular and mortality risks [12, 13]. Increased lifetime exposure to hyperglycemia and insulin resistance is likely to be associated with higher risks of vascular complications [14] and microvascular disease of the central nervous system [15]. Younger age at T2DM onset may likely pose relatively greater excess dementia risks than older age at onset. However, studies examined the association of age at diagnosis of diabetes with cognitive impairment and/or dementia often treat individuals free of or never developed diabetes at a specific age being the reference group [16–18]. Although the prevalence of T2DM has risen, few studies have examined the association of age at diabetes diagnosis with dementia explicitly in individuals with T2DM.

The growth in T2DM has been influenced by escalating rates of obesity in middle-aged and older adults. Obesity and diabetes are significant risk factors for developing dementia because key mediators of dementia progression are inflammation, oxidative stress, and cerebrovascular disease within the brain that contribute to neuronal dysfunction and death [19, 20]. It is hypothesized that obesity is not a primary risk factor for diminished cognitive functioning, but exacerbates the effects of cardiovascular risk factors (e.g., hypertension, diabetes, high cholesterol, and smoking) on cognition [21]. However, studies investigated the effect of obesity on the association between age at diagnosis of T2DM and dementia are limited.

We aimed to examine the association between age at diagnosis of T2DM and the risk of dementia and the moderation effect of obesity on this association among adults aged ≥50 with previously diagnosed T2DM. We hypothesized that 1) a younger age of T2DM diagnosis would be associated with a higher risk of dementia than an older age of diagnosis; 2) the association will be more prominent among diabetic individuals who are obese.

## Methods

### Data sources and sample

Data were from the Health and Retirement Survey (HRS) 2002–2016 and its 2003 Diabetes Mail-Out Survey. The HRS uses a multi-stage area probability sample design with oversampling of Black and Hispanic individuals and residents of Florida. This design ensures that the sample is representative of the US population over age 50. HRS interviews were conducted by trained interviewers who are prepared to accommodate the needs of older adults, including those with sensory or cognitive limitations. The 2003 Diabetes Mail-Out Study was conducted among 2,350 respondents who reported having diabetes in the 2002 wave; 1,901 completed the survey (80.9% response rate) [22]. The survey collected information on age at diagnosis of diabetes, diabetes care, and self-management. Respondents received a self-administered HbA1c fingerstick kit with detailed instructions, and returned samples were analyzed in a central laboratory following standardized protocols [23]. The HRS uses a comprehensive questionnaire that typically takes about two hours to complete, though the duration can vary depending on the participant’s circumstances and the specific modules included in each wave. The core survey contains approximately 400–500 questions. The 2003 Diabetes Mail-Out Survey, which was a supplementary questionnaire focused specifically on diabetes, contained approximately 30 questions and took about 20–30 minutes to complete on average. A total of 1,233 participants successfully completed the HbA1c blood spot samples. The HRS data used for this analysis are de-identified and publicly available.

**Fig 1:**
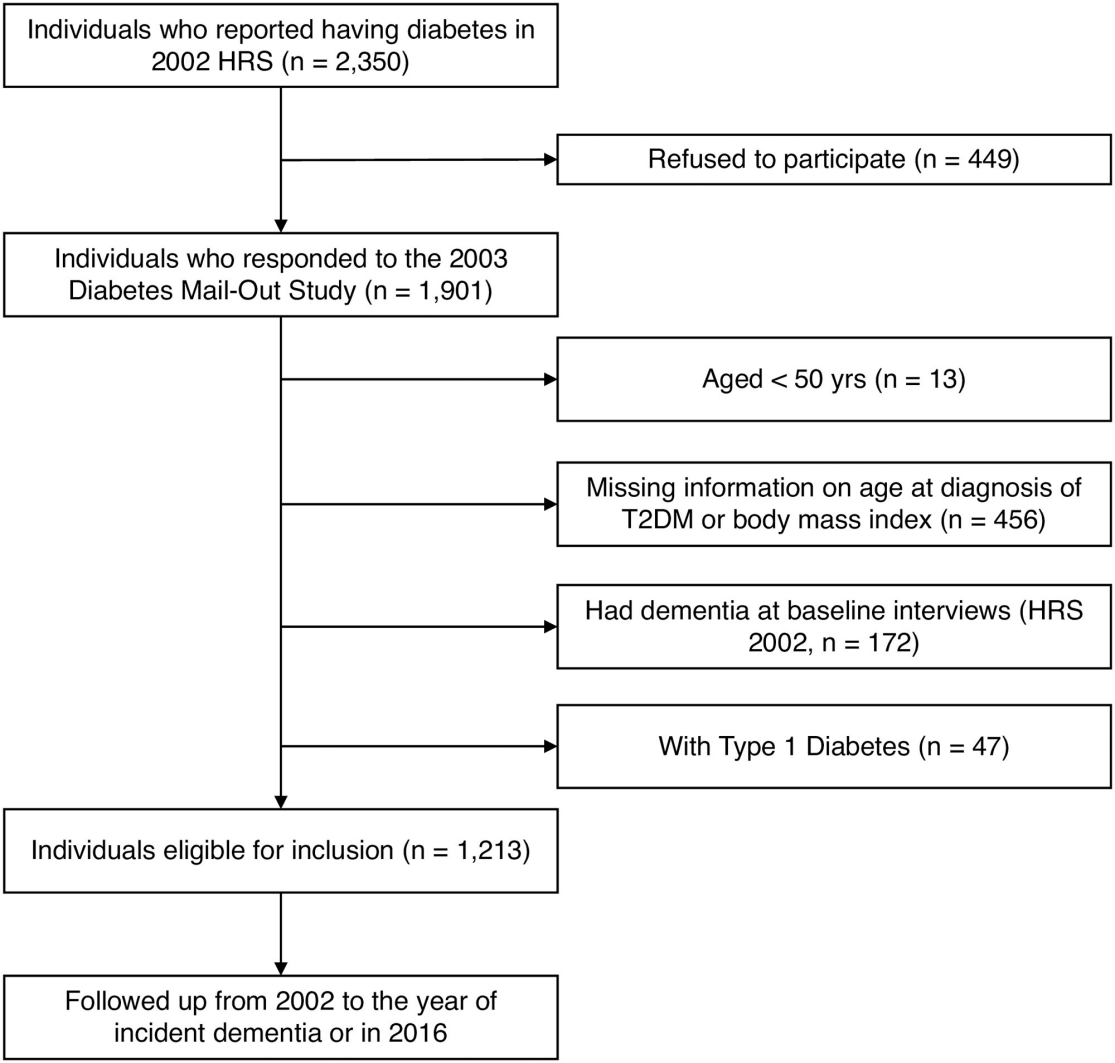
Flowchart of the sample selection.

We linked the 2003 Diabetes Study to 2002–2016 HRS data. Among the 1,901 HRS respondents from 2002 to 2016, we excluded individuals who were 1) chronological age <50 years (n = 13); 2) with missing information on age at diagnosis of diabetes or BMI (n = 456); and 3) respondents or their proxies answered ‘yes’ to the question ‘Has a doctor ever told you that you have Alzheimer’s disease or other dementia’ or score 0–7 on the cognitive assessment using the modified version of Telephone Interview for Cognitive Status in HRS 2002 (n = 172). Individuals with type 1 diabetes were excluded (n = 47), given that type 1 diabetes typically occurs in children and young adults and the different pathology with T2DM. The analysis cohort thus consisted of 1,213 participants with T2DM who had complete measures on age at the diagnosis of T2DM, BMI, cognitive functioning, and a follow-up period to the end of 2016 (14 years maximum) with time-to-events being new-onset dementia (sample selection flowchart in Fig 1).

#### Ethics statement

The HRS protocol was approved by the Institutional Review Board (IRB #HUM00061128) at the University of Michigan, and all participants provided written informed consent. This project is a secondary analysis of the HRS (https://hrs.isr.umich.edu/about), which uses completely deidentified public datasets. As this study did not involve direct human participants and utilized only publicly available data, per the Common Rule (45 CFR §46), local IRB and approval was not required.

### Measurement

#### Age at diabetes diagnosis and obesity

In the HRS 2003 module, the age at diagnosis of T2DM was measured by asking each participant, “At what age were you told by a physician that you had diabetes.” We categorized it as age at diagnosis of T2DM <50 years, 50–59 years, 60–69 years, and ≥70 years. BMI was calculated as the self-reported body weight divided by the square of the self-reported body height (kg/m^2^). Obesity was defined as a BMI of ≥30.0 kg/m^2^, and individuals with a BMI <30 kg/m^2^ are classified as non-obese.

#### Dementia classification

HRS assessed cognitive function via the modified version of the Telephone Interview for Cognitive Status (TICS). We followed previous studies [24, 25] of getting a score by summing the 27 cognitive items: immediate and delayed recall of a list of 10 words (1 point for each), five trials of serial 7s (i.e., subtract 7 from 100, and continue subtracting 7 from each next number for a total of five trials, 1 point for each trial), and backward counting (2 points). The summary score ranges from 0 (impaired functioning) to 27 (high functioning). Respondents whose scores were 0–6 were classified as having dementia; those whose scores were 7–27 were classified as not having dementia [24]. The cutoff point has been validated against the prevalence of dementia in the Aging, Demographics, and Memory Study (an HRS sub-study that applies neuropsychological and clinical assessment of dementia) by correctly classifying 78% of HRS respondents as having dementia or not [24, 26].

#### Covariates

Covariates at baseline were controlled based on previous studies [5, 16, 21]. Sociodemographic variables included age, sex, race/ethnicity (Hispanic, non-Hispanic Black, non-Hispanic White, and others), education attainment (<high school, high school diploma, some/completed college, and graduate degree), and annual household income ($). Health behaviors variables included current smoker (yes/no) and participation in regular exercise programs in the past two weeks (yes/no). Health-related variables include HbA1c, BMI, and comorbid conditions (having one or more self-reported hypertension, heart disease, stroke, arthritis, cancer, and lung disease). Diabetes medication covariates include insulin use and oral hypoglycemic medication use, using self-reported answers.

### Statistical analyses

Continuous variables are presented as means ± SDs or medians (IQRs), and categorical variables are presented as percentages (%). The characteristics according to age at diagnosis of T2DM were calculated using ANOVA or a rank-sum test for continuous variables and the χ^2^ test for categorical variables.

Respondents were censored at the date of record of dementia, death, or 2016 HRS survey, whichever came first. Cox proportional hazard models were used to estimate the association of age at diagnosis of T2DM with dementia, with the diagnosed ≥70 years group as the reference. The hazard ratios (HRs) and 95% confidence intervals (CIs) for incident dementia were estimated. Model 1 was unadjusted; Model 2 was adjusted for sociodemographic variables; Model 3 was further adjusted for health behaviors variables; Model 4 was further adjusted for health characteristics; Model 5 was further adjusted for diabetes medication covariates. Additionally, the cross-products between age at diagnosis of T2DM and obesity were added as interaction terms in Model 6. Missing values accounted for <2.0% of all the covariates, except for HbA1c (18.2%). Individuals who did not develop dementia, died, or were lost to follow-up were censored at their last assessment. The Cox proportional hazard assumption was checked using a log (-log (Survival)) plot.

We further examined the combined effects of obesity and age at diagnosis of T2DM on the risk of dementia. Following previous studies [13, 16] that examined the association between age at diabetes diagnosis and morbidity and mortality, and to account for the distribution of the age at T2DM diagnosis of our participants, we categorized participants into four groups based on a combination of age at diagnosis (<50 years or ≥50 years) and obesity (obese or non-obese).

To test the robustness of the results, we conducted several sensitivity analyses: (1) age at diagnosis of T2DM was analyzed as a continuous variable, and the association between every one-year younger age at diagnosis and dementia was estimated; (2) to evaluate the influence of glycemic control and degree of T2DM severity, we compared the participants a) with HbA1c level ≥7.0% (53 mmol/mol) vs. those with HbA1c level <7.0% and b) participants did not use insulin vs. use insulin; (3) we iteratively imputed missing information related to covariates using random forest multivariate imputation [27]. Imputation was implemented using the *missforest* R package.

All analyses were weighted to adjust for the complex sample design of the HRS, the differential probability of selection, and nonresponse to the 2003 Diabetes Mail-Out Survey and HbA1c testing. All analyses were performed using Stata/SE 15.1 (StataCorp LP, College Station, TX) and R version 3.5.1 (R Program for Statistical Computing). Significance was indicated at two-sided *P* < .05.

## Results

### Characteristics of the participants

Characteristics of the 1,213 participants according to age at diagnosis of T2DM were presented in Table 1. Overall, 216 (17.8%) participants developed dementia during a medium of 10-year [IQR, 6–14] follow-up. For age at T2DM diagnosis, 262 participants are at <50 years; 378, at 50–59 years; 419, at 60–69 years; and 154 at ≥70 years. On average, participants with younger age at diagnosis were more likely to be female, racial/ethnic minoritized, current smokers, have higher BMI, and poorer glycemic control (all *P* < .01). Besides, participants with younger age at diagnosis had higher insulin use, and lower oral hypoglycemic medications use (all *P* < .05).

**Table 1:** Baseline characteristics according to age at diagnosis of T2DM (n = 1,213).

Characteristics	Total sample	Age at the Diagnosis of Type 2 Diabetes Mellitus (years)				
		≥70 (n = 154)	60–69 (n = 419)	50–59 (n = 378)	<50 (n = 262)	*P* value^a^
**Chronological age, mean (SD), years**	68.8 (8.2)	79.5 (4.9)	71.1 (6.1)	65.0 (6.9)	64.5 (7.5)	< .001
**Female, %**	54.8	59.7	48.2	56.4	60.3	< .01
**Race/ethnicity**, %						< .01
Non-Hispanic White	70.8	78.6	75.9	68.8	61.3	
Non-Hispanic Black	16.1	11.0	12.9	17.2	22.6	
Hispanic	10.6	7.8	9.6	11.6	12.3	
Others	2.5	2.6	1.7	2.4	3.8	
**Education attainment**, %						.628
<High school	30.0	27.9	30.1	27.5	34.7	
High school diploma	34.4	36.4	33.9	35.7	32.1	
Some/completed college	26.9	27.3	25.5	29.4	25.6	
Graduate degree	8.7	8.4	10.5	7.4	7.6	
**Household income**^b^ **($), median (IQR)**	29,915 (16,090–53,299)	27,470 (15,300–40,800)	28,524 (16,860–49,520)	34,240 (19,000–58,000)	27,516 (14,160–55,024)	< .05
**Current smoker**, %	10.3	3.3	9.7	13.5	15.0	< .01
**Have regular physical exercise, %**	55.2	60.1	57.6	56.4	54.1	.668
**BMI, mean (SD), kg/m** ^**2**^	30.3 (6.2)	28.1 (5.6)	29.5 (5.2)	31.4 (6.7)	31.3 (6.5)	< .001
**Obesity, %**	46.7	30.5	43.7	53.4	51.5	< .001
**HbA1c, mean (SD), %**	7.2 (1.4)	7.0 (1.2)	7.0 (1.2)	7.3 (1.5)	7.6 (1.6)	< .001
**Insulin use, %**	23.8	8.1	15.1	22.3	49.2	< .001
**Oral hypoglycemic medication use, %**	75.6	78.5	75.2	79.2	69.4	< .05
**At least one comorbid condition** ^c^ **, %**	34.7	44.8	39.6	31.5	25.6	< .001

Note:

Abbreviations: SD, standard deviation; BMI, body mass index (calculated as weight in kilograms divided by height in meters squared); HbA1c, glycated hemoglobin.

^a^ *P* values are from ANOVA or rank sum test (for household income) for continuous variables and the χ^2^ test for categorical variables.

^b^ Group comparison was performed on log transformed data.

^c^ Have one or more of comorbid condition: self-reported hypertension, heart disease, stroke, arthritis, cancer, and lung disease.

### Age at diagnosis of T2DM and the risk of dementia

We examined the association between age at diagnosis of T2DM and the risk of dementia in the total sample (Table 2). In unadjusted Model 1, compared with participants diagnosed with T2DM ≥70 years, younger ages at diagnosis (diagnosed 60–69, 50–59, <50) were associated with 88%, 81%, and 201% increased risks for dementia, respectively (*P* for trend < .001). Further adjustment for sociodemographics, health behaviors, health status, and diabetes medication did not change the estimates significantly. Compared with individuals diagnosed ≥70 years, the fully-adjusted HRs (95% CIs) of dementia for those diagnosed aged 60–69, 50–59, and <50 years are 1.70 (1.03–2,80), 1.72 (1.06–2.79), and 1.90 (1.14–3.18), respectively. The HRs for dementia tended to increase with younger age at diagnosis (*P* for trend < .001).

**Table 2:** Hazards ratio (95% CIs) for dementia risks according to age at diagnosis of T2DM (HRS 2002–2016).

Age at Diagnosis of T2DM (years)	No of dementia cases/No. of participants (incidence, %)	Model 1	Model 2	Model 3	Model 4	Model 5	Model 6
		Hazards Ratio (95% Confidence Intervals)					
≥70 (Ref.)	24/154 (15.6)	1.00	1.00	1.00	1.00	1.00	1.00
60–69	71/419 (16.9)	1.88 (1.10, 3.21)*	1.77 (1.03, 3.02)*	2.11 (1.21, 3.67)**	1.92 (1.10, 3.37)*	1.70 (1.03, 2.80)*	1.83 (0.92, 3.63)
50–59	50/378 (13.2)	1.81 (1.25, 2.62)**	2.01 (1.24, 3.26)**	2.19 (1.34, 3.60)**	1.99 (1.20, 3.30)**	1.72 (1.06, 2.79)*	1.45 (1.11, 1.89)**
<50	71/262 (27.1)	3.01 (1.80, 5.03)***	2.51 (1.50, 4.19)***	2.64 (1.57, 4.44)***	2.16 (1.28, 3.66)**	1.90 (1.14, 3.18)*	1.66 (1.27, 2.17)**
*P* for trend		< .001	< .001	< .001	< .01	< .01	.287
**Age at diagnosis by obesity interaction (Ref. ≥70 & non-obese)**							
60–69 × Obese							1.09 (0.35, 3.42)
50–59 × Obese							1.82 (1.08, 3.07)*
<50 × Obese							1.93 (1.21, 1.75)***

Note:

Analyses using Cox proportional hazards model when the outcome was incident dementia, and were weighted to adjust for the complex sample design of the Health and Retirement Study 2003 Diabetes Mail-Out Survey.

Trend test performed using the median values for each age at the diagnosis of T2DM category.

Model 1, unadjusted.

Model 2, adjusted for age, sex, race/ethnicity, income, and education.

Model 3, further adjusted for smoking and physical exercise.

Model 4, further adjusted for HbA1c, body mass index, and comorbid conditions.

Model 5, further adjusted for insulin use and oral hyperglycemic medication use.

Model 6, further added the interaction terms between age at diagnosis of T2DM and obesity.

*P < .05,

**P < .01,

***P < .001.

### Moderation effect of obesity

Of 1,213 participants, 567 (46.7%) were obese. Significant interactions between obesity and age at diagnosis of T2DM were obtained for the risk of dementia (*P* < .05 for the interactions between those diagnosed at 50–59 years and those at <50 years with obesity). We further explored the combined effects of obesity and age at diagnosis of T2DM on the risk of dementia (Table 3). Results from the fully-adjusted Cox model revealed that compared with the reference group (non-obese and diagnosed ≥50 years), those with obesity and diagnosed <50 years had the highest risk of dementia (HR, 3.05; 95% CI 1.23–7.56).

**Table 3:** Hazard ratio (95% CI) for dementia risks according to the combination of obesity and age at diagnosis of T2DM (HRS 2002–2016).

Age at Diagnosis of T2DM (years) and Obesity Status	No of dementia cases/No. of participants (incidence, %)	Model 1	Model 2	Model 3	Model 4
		Hazards Ratio (95% Confidence Intervals)			
≥50 years and non-obese (Ref.)	83/519 (16.0)	1.00	1.00	1.00	1.00
≥50 years and obese	62/432 (14.4)	2.10 (0.84, 5.26)	1.73 (0.67, 4.45)	1.79 (0.64, 5.02)	1.79 (0.64, 4.99)
<50 years and non-obese	33/127 (26.0)	2.56 (1.16, 5.66)*	2.50 (1.12, 5.54)*	2.74 (1.21, 6.22)*	2.21 (1.02, 4.79)*
<50 years and obese	38/135 (28.1)	3.30 (1.16, 5.65)**	2.99 (1.34, 6.70)**	3.50 (1.42, 8.64)**	3.05 (1.23, 7.56)*

Note:

Analyses using Cox proportional hazards model when the outcome was incident dementia, and were weighted to adjust for the complex sample design of the Health and Retirement Study 2003 Diabetes Mail-Out Survey.

Model 1: adjusted for sociodemographic variables (age, sex, race/ethnicity, income, and education).

Model 2: further adjusted for health behaviors (smoking and physical exercise).

Model 3: further adjusted for health-related variables (HbA1c and comorbid conditions).

Model 4: further adjusted for diabetes medication (insulin and oral hypoglycemic medication).

*P < .05,

**P < .01,

***P < .001.

### Sensitivity analyses

When age at T2DM diagnosis was modeled continuously, each one-year younger age at diagnosis was significantly associated with a 1.9% increased risk of dementia (HR, 1.02; 95% CI, 1.01–1.03; *P* < .05). A significant interaction was detected between one-year younger age at diagnosis of T2DM and obesity (*P* = .022). S1 and S2 Tables summarize the association of age at diagnosis of T2DM and risks for dementia when stratified by glycemic control (HbA1c≥7.0% vs. <7.0) and insulin use (yes vs. no). The findings are consistent with our primary results that younger age at T2DM diagnosis is associated with a higher risk of dementia, regardless of glycemic control and insulin use. The difference in the distribution of the participants’ characteristics before and after multiple imputations is presented in S3 Table. The results using the imputed data were consistent with the increased HRs with younger age at diagnosis in the primary results, and the moderation effects of obesity are still significant (S4 Table).

## Discussion

In this 14-year prospective cohort study of adults aged ≥50 with T2DM, we found an increasing trend of younger age at diagnosis of T2DM and a higher risk of dementia, and obesity moderated the associations. Incremental dementia risk associated with T2DM was attenuated by the increases in the age at diagnosis of T2DM. The dementia risk was even more markedly elevated in obese individuals with T2DM diagnosed at a younger age. Our findings underscore the potential consequences of temporal trends in obesity and diabetes and suggest the need for interventions targeting dementia prevention among adults with T2DM, particularly for those with the disease onset <50 years old.

Emerging evidence suggests that younger age at diagnosis of diabetes is associated with increased risks of cognitive health outcomes [16–18]. Results from the Swedish Twin Registry study, based on a population-based sample of adults aged ≥65 years, suggest that the risk effect of mid-life diabetes (onset age <65 years) on dementia was stronger than late-life diabetes (onset age ≥65 years), and these effects are independent of genetic and early-life environmental factors [18]. In another study of older adults without dementia, those with a T2DM diagnosis in midlife had more brain volume loss, lower global cognition, and lower executive function in later life than those without a T2DM diagnosis in midlife [28]. Although these studies have demonstrated increased risks for cognitive health outcomes in adults with younger onset of diabetes, very few have examined the effect of age of T2DM diagnosis in a dose-response pattern. Careful analysis of age of T2DM diagnosis in the present study, showed a graded association between age at diagnosis of T2DM and dementia risk.

The mechanisms that link younger diabetes diagnosis to dementia are not comprehensively understood. Poorer glycemic control and more vascular complications in individuals with younger age at diagnosis have been reported in previous studies [11, 29, 30]. Individuals with diabetes who are younger at diagnosis may have a more physiologically aggressive form of diabetes, with worse glycemic control, beta cell dysfunction, and insulin resistance. This explanation is supported by a national cohort study from the Swedish National Diabetes Register, which found that adults with the younger onset of T2DM display a more adverse lipid profile, have higher HbA1c, and have a faster deterioration in glycemic control compared with individuals who develop T2DM later in life [11]. Vascular dysfunction, poor glycemic control, and insulin resistance are risk factors for cognitive impairment [15, 31]. Notably, our study’s sensitivity analysis stratified by glycemic control and insulin use did not significantly change the association between age at diagnosis and dementia, suggesting that other pathophysiologic mechanisms are involved.

Our study found that obesity modifies the association between age at diagnosis and dementia among individuals with T2DM. While there is limited evidence regarding the moderation effect of obesity on the association between age at diagnosis of diabetes and dementia, studies have demonstrated that BMI changes interfere with the association between diabetes and diminished cognitive functioning [32–34]. Epidemiologic studies have suggested that obesity combined with diabetes is associated with poorer cognitive function and an elevated risk of Alzheimer’s disease and dementia in an additive interaction manner [32, 35]. A South Korean nationwide cohort study reported that excessive weight gain significantly increases the risk of all-cause dementia in middle-aged and older individuals with T2DM [34].

Evidence suggests that inflammation and insulin resistance caused by obesity and diabetes are associated with amyloid and Alzheimer’s disease-pattern neurodegeneration [36]. Individuals with younger ages at diagnosis of diabetes are less likely to maintain strict glycemic control and lose weight, which can lead to hypoglycemia and worse lipid profiles associated with dementia [11]. Obesity is a more common feature of early-onset T2DM than late-onset T2DM [11, 12]. An alternative explanation may be that diabetes develops in the context of other vascular risk factors (e.g., obesity) that are often present long before the onset of diabetes. When diabetes eventually develops, hyperglycemic damage could develop on top of the damage already done by these vascular risk factors in the preceding years.

Our findings highlight the importance of age at diagnosis in the management, screening, and preventative strategies of T2DM. Targeting obesity through behavioral interventions may be promising, and a better understanding of the mechanisms that underlie the association between diabetes and dementia pathology may aid in identifying populations that would benefit from obesity-modifying therapies. Hence, treatment could focus on shared mechanisms of diabetes and obesity, such as insulin resistance. In addition, multifactorial treatment targeting multiple vascular risk factors may be a fruitful approach to dementia prevention.

Several limitations should be acknowledged. First, T2DM was self-reported at baseline and our study related to the use of phone interviews for data collection. While the HRS employs rigorous protocols to verify participant identity and ensure data integrity during phone interviews. the possibility of measurement errors and information bias cannot be completely eliminated. Future studies might benefit from incorporating objective measurements and additional verification methods for phone interviews to further enhance data validity. Second, our study is limited by the availability of diabetes control data only from the HRS 2003 Diabetes Mail-Out Survey. We acknowledge that diabetes management may change over time, potentially influencing dementia risk. Future studies incorporating repeated measures of diabetes control could provide more nuanced insights into their long-term effects on cognitive outcomes in individuals with T2DM. Third, the measure of dementia is based on cognitive tests rather than clinical diagnosis. Although previous research using HRS has demonstrated that cognitive tests correctly classify 74% of HRS participants into clinical diagnosis categories of dementia [26], the misclassification bias cannot be ignored. Fourth, obesity is often the cornerstone that leads to the development of T2DM, but we could not consider the duration or timing of exposures to obesity in this dataset. Fifth, several types of dementia (e.g., Alzheimer’s disease, vascular dementia, Lewy-body dementia) relate to diabetes through different mechanisms. We cannot discriminate among these types in this study. For future studies, the underlying mechanisms of the association between age at diagnosis of diabetes and types of dementia need to be clarified. In addition, future studies are needed to include information on lipoprotein, insulin levels, and waist-hip ratio to elucidate the complex associations between T2DM, obesity, and dementia.

## Conclusion

This 14-year prospective cohort study for individuals with T2DM suggests a graded association between age at diagnosis of diabetes and dementia, particularly in individuals with obesity. This study provides a framework for future studies to explore the mechanisms and pathways of the diabetes-dementia relationship. Future studies are needed to address the complexity of T2DM, obesity, and dementia relationship. Interventions specifically targeting obesity may be more effective in preventing dementia for adults with younger age at diagnoses of T2DM.

## Supporting information

S1 Table Hazard ratio (95% CI) for dementia risks according to age at diagnosis of T2DM and glycemic control. (DOCX)

S2 Table Hazard ratio (95% CI) for dementia risks according to age at diagnosis of T2DM and insulin use. (DOCX)

S3 Table Distribution of participants’ characteristics before and after multiple imputation. (DOCX)

S4 Table Hazard ratio (95% CI) for dementia risks using the imputed dataset (HRS 2002–2016). (DOCX)
