# Supplementary material for: Age at diagnosis of diabetes, obesity, and the risk of dementia among adult patients with type 2 diabetes
Source: PLoS One. 2024 Nov 13;19(11):e0310964. doi: 10.1371/journal.pone.0310964 (PMC11559992; doi:10.1371/journal.pone.0310964)
Supplement: S1 Table — (DOCX) [file pone.0310964.s001.docx]

**S1 Table. Hazard ratio (95% CI) for dementia risks according to age at diagnosis of T2DM and glycemic control.**

| **Age at the Diagnosis of Type 2 Diabetes (years)** | **Glycemic control** | |
| --- | --- | --- |
|  | **HbA1c ≥7.0% (n = 555)** | **HbA1c <7.0% (n = 438)** |
| **≥70** | 1.00 (Ref.) | 1.00 (Ref.) |
| **60-69** | 1.70 (1.03, 2.80)^*^ | 1.69 (0.98, 2.90) |
| **50-59** | 1.72 (0.91, 3.25) | 1.67 (0.96, 2.91) |
| **<50** | 1.90 (1.14, 3.18)^**^ | 2.09 (1.23, 3.54)^**^ |
| ***P* for trend** | < .05 | < .05 |
| **Each year earlier** | 1.01 (1.00-1.02)^*^ | 1.03 (1.01, 1.04)^**^ |

Note:

Analyses using Cox proportional hazards model when the outcome was incident dementia. Models were adjusted for sociodemographic variables (age, sex, race/ethnicity, income, and education), health behaviors (smoking and physical exercise), health-related variables (body mass index, and comorbid conditions), and diabetes medication (insulin use and oral hypoglycemic medication use).
