# Supplementary material for: Age at diagnosis of diabetes, obesity, and the risk of dementia among adult patients with type 2 diabetes
Source: PLoS One. 2024 Nov 13;19(11):e0310964. doi: 10.1371/journal.pone.0310964 (PMC11559992; doi:10.1371/journal.pone.0310964)
Supplement: S2 Table — (DOCX) [file pone.0310964.s002.docx]

**S2 Table. Hazard ratio (95% CI) for dementia risks according to age at diagnosis of T2DM and insulin use.**

| **Age at the Diagnosis of Type 2 Diabetes (years)** | **Insulin user** | |
| --- | --- | --- |
|  | **Yes (n = 281)** | **No (n = 902)** |
| **≥70** | 1.00 (Ref.) | 1.00 (Ref.) |
| **60-69** | 2.07 (1.06, 4.04)^*^ | 1.29 (0.98, 2.90)^*^ |
| **50-59** | 1.32 (0.66, 2.64) | 0.92 (1.07, 2.93) |
| **<50** | 2.04 (0.94, 4.43) | 2.43 (1.23, 3.54)^**^ |
| ***P* for trend** | .562 | <.05 |
| **Each year earlier** | 1.02 (1.01, 1.03)^*^ | 1.03 (1.01, 1.04)^**^ |

Note:

Analyses using Cox proportional hazards model when the outcome was incident dementia. Models were adjusted for sociodemographic variables (age, sex, race/ethnicity, income, and education), health behaviors (smoking and physical exercise), and health-related variables (HbA1c, body mass index, and comorbid conditions).
