# Supplementary material for: Age at diagnosis of diabetes, obesity, and the risk of dementia among adult patients with type 2 diabetes
Source: PLoS One. 2024 Nov 13;19(11):e0310964. doi: 10.1371/journal.pone.0310964 (PMC11559992; doi:10.1371/journal.pone.0310964)
Supplement: S3 Table — (DOCX) [file pone.0310964.s003.docx]

**S3 Table. Distribution of participants’ characteristics before and after multiple imputation.**

| **Characteristics** | **Not imputed dataset** | **Imputed dataset** |
| --- | --- | --- |
| **Age, mean (SD), years** | 68.8 (8.2) | 68.7 (8.0) |
| **Female, %** | 54.8 | 54.8 |
| **Race/ethnicity**, % |  |  |
| Non-Hispanic White | 70.8 | 70.8 |
| Non-Hispanic Black | 16.1 | 16.2 |
| Hispanic | 10.6 | 10.7 |
| Others | 2.5 | 2.4 |
| **Education attainment**, % |  |  |
| <High school | 30.0 | 29.9 |
| High school diploma | 34.4 | 34.3 |
| Some/completed college | 26.9 | 25.9 |
| Graduate degree | 8.7 | 9.9 |
| **Household income**^a^ **($), median (IQR)** | 29,915 (16,090-53,299) | 29,984 (16,072-53,728) |
| **Current smoker**, % | 10.3 | 10.3 |
| **Have regular physical exercise, %** | 55.2 | 55.4 |
| **BMI, mean (SD), kg/m^2^** | 30.3 (6.2) | 30.2 (6.0) |
| **Mean age at diagnosis of T2DM (SD), years** | 56.5 (13.2) | 56.1 (12.8) |
| **HbA1c, mean (SD), %** | 7.2 (1.4) | 7.2 (1.2) |
| **Insulin use, %** | 23.8 | 25.3 |
| **Oral hypoglycemic medication use, %** | 75.6 | 74.4 |
| **At least one comorbid condition**^b^**, %** | 34.7 | 34.0 |

Note:

Abbreviations: SD, standard deviation; BMI, body mass index (calculated as weight in kilograms divided by height in meters squared); HbA1c, glycated hemoglobin.

^a^ Log transformed data.

^b^ Have one or more of comorbid condition: self-reported hypertension, heart disease, stroke, arthritis, cancer, and lung disease.
