# Supplementary material for: Age at diagnosis of diabetes, obesity, and the risk of dementia among adult patients with type 2 diabetes
Source: PLoS One. 2024 Nov 13;19(11):e0310964. doi: 10.1371/journal.pone.0310964 (PMC11559992; doi:10.1371/journal.pone.0310964)
Supplement: S4 Table — (DOCX) [file pone.0310964.s004.docx]

**S4 Table. Hazard ratio (95% CI) for dementia risks using the imputed dataset (HRS 2002-2016).**

| **Age at Diagnosis of T2DM (years)** | **Model 1** | **Model 2** | **Model 3** | **Model 4** | **Model 5** |
| --- | --- | --- | --- | --- | --- |
|  | Hazards Ratio (95% Confidence Intervals) | | | | |
| ≥70 (Ref, n = 154) | 1.00 | 1.00 | 1.00 | 1.00 | 1.00 |
| 60-69 (n = 419) | 1.71 (1.01, 2.91)^*^ | 2.03 (1.17, 3.51)^**^ | 1.90 (0.95, 3.79) | 1.87 (0.99, 3.53) | 1.77 (0.67, 3.72) |
| 50-59 (n = 378) | 1.99 (1.24, 3.19)^*^ | 2.17 (1.34, 3.51)^**^ | 1.87 (1.21, 2.89)^*^ | 1.92 (1.23, 2.99)^*^ | 1.36 (1.05, 1.76)^*^ |
| <50 (n = 262) | 2.43 (1.47, 4.00)^***^ | 2.59 (1.56, 4.31)^***^ | 2.09 (1.40, 3.11)^***^ | 2.01 (1.31, 3.08)^***^ | 1.46 (1.08, 1.97)^**^ |
| *P* for trend | < .001 | < .001 | < .05 | < .01 | .127 |
| **Age at diagnosis by obesity interaction (Ref. ≥70 & non-obese)** |  |  |  |  |  |
| 60-69 × Obese |  |  |  |  | 1.81 (0.74, 4.43) |
| 50-59 × Obese |  |  |  |  | 2.38 (1.05, 5.39)^**^ |
| <50 × Obese |  |  |  |  | 2.87 (1.18, 6.97)^**^ |

Note:

Analyses using Cox proportional hazards model when the outcome was incident dementia.

Trend test performed using the median values for each age at the diagnosis of T2DM category.

Model 1, adjusted for age, sex, race/ethnicity, income, and education.

Model 2, further adjusted for smoking and physical exercise.

Model 3, further adjusted for HbA1c, body mass index, and comorbid conditions.

Model 4, further adjusted for insulin use and oral hyperglycemic medication use.

Model 5, further added the interaction terms between age at diagnosis of T2DM and obesity.

^*^*P* < .05, ^**^*P* < .01, ^***^*P* < .001.
